# Using blood cytokine measures to define high inflammatory biotype of schizophrenia and schizoaffective disorder

**DOI:** 10.1186/s12974-017-0962-y

**Published:** 2017-09-18

**Authors:** Danny Boerrigter, Thomas W. Weickert, Rhoshel Lenroot, Maryanne O’Donnell, Cherrie Galletly, Dennis Liu, Martin Burgess, Roxanne Cadiz, Isabella Jacomb, Vibeke S. Catts, Stu G. Fillman, Cynthia Shannon Weickert

**Affiliations:** 1 Neuroscience Research Australia, and Schizophrenia Research Institute, Barker Street, Randwick, New South Wales 2031 Australia; 2 School of Psychiatry, University of New South Wales, Kensington, New South Wales Australia; 3 Discipline of Psychiatry, School of Medicine, The University of Adelaide, Adelaide, South Australia Australia; 4 Northern Adelaide Local Health Network, Adelaide, South Australia Australia; 5 Ramsay Health Care (SA) Mental Health, Adelaide, South Australia Australia

**Keywords:** Cytokines, Inflammation, Periphery, Schizophrenia, Biotype, Gene expression, Protein

## Abstract

**Background:**

Increases in pro-inflammatory cytokines are found in the brain and blood of people with schizophrenia. However, increased cytokines are not evident in all people with schizophrenia, but are found in a subset. The cytokine changes that best define this subset, termed the “elevated inflammatory biotype”, are still being identified.

**Methods:**

Using quantitative RT-PCR, we measured five cytokine mRNAs (IL-1β, IL-2 IL-6, IL-8 and IL-18) from peripheral blood of healthy controls and of people with schizophrenia or schizoaffective disorder (*n* = 165). We used a cluster analysis of the transcript levels to define those with low and those with elevated levels of cytokine expression. From the same cohort, eight cytokine proteins (IL-1β, IL-2, IL-6, IL-8, IL-10, IL-12, IFNγ and TNFα) were measured in serum and plasma using a Luminex Magpix-based assay. We compared peripheral mRNA and protein levels across diagnostic groups and between those with low and elevated levels of cytokine expression according to our transcription-based cluster analysis.

**Results:**

We found an overall decrease in the anti-inflammatory IL-2 mRNA (*p* = 0.006) and an increase in three serum cytokines, IL-6 (*p* = 0.010), IL-8 (*p* = 0.024) and TNFα (*p* < 0.001) in people with schizophrenia compared to healthy controls. A greater percentage of people with schizophrenia (48%) were categorised into the elevated inflammatory biotype compared to healthy controls (33%). The magnitude of increase in IL-1β, IL-6, IL-8 and IL-10 mRNAs in people in the elevated inflammation biotype ranged from 100 to 220% of those in the non-elevated inflammatory biotype and was comparable between control and schizophrenia groups. Blood cytokine protein levels did not correlate with cytokine mRNA levels, and plasma levels of only two cytokines distinguished the elevated and low inflammatory biotypes, with IL-1β significantly increased in the elevated cytokine control group and IL-8 significantly increased in the elevated cytokine schizophrenia group.

**Conclusions:**

Our results confirm that individuals with schizophrenia are more likely to have elevated levels of inflammation compared to controls. We suggest that efforts to define inflammatory status based on peripheral measures need to consider both mRNA and protein measures as each have distinct advantages and disadvantages and can yield different results.

**Electronic supplementary material:**

The online version of this article (10.1186/s12974-017-0962-y) contains supplementary material, which is available to authorized users.

## Background

While the ubiquity of neuroinflammation in schizophrenia has been challenged [1], it is clear that elevated brain cytokine expression is found in a subset of people with schizophrenia [2–4]. It is possible that individuals with elevated brain cytokine expression belong to a distinct inflammatory “biotype”, in which biotype is defined as a subset of individuals identified as being similar based on levels of a biological marker or a combination of biological markers [5]. Previous work in our laboratory has shown that this apparent inflammatory biotype of schizophrenia based on cortical cytokine expression may also be identified in a living clinical cohort based on peripheral cytokine expression [6] with ~ 40% of people with schizophrenia classified in the elevated inflammatory biotype based on measures from the post-mortem brain or from peripheral white blood cells. Recent studies of major depression and bipolar affective disorder by Andrew Miller and colleagues have demonstrated the utility of stratifying individuals by inflammatory status; they found that those who responded to anti-inflammatory treatment had transcriptional changes in the innate immune system [7]. In clinical trials with individuals with major depression, they showed significant improvement in symptoms [8] after an anti-inflammatory (infliximab) treatment and improved quality of sleep [9], but only in those individuals with elevated inflammation defined as elevated C-reactive protein (CRP) levels. Such stratification may also be helpful in schizophrenia. For example, we have found that the high inflammatory biotype of schizophrenia is more likely to display lower levels of inhibitory interneuron markers, greater cortical grey matter reduction and signs of astrogliosis [2, 10, 11]. Identifying this subgroup of individuals may help to determine who could respond best to novel anti-inflammatory treatments.

In our previous studies, we found a significantly higher proportion of people with schizophrenia versus healthy controls with elevated cytokines and an inflammation-associated reduction in brain volume [6, 11]. While we found decreased cognitive ability in people with schizophrenia with elevated cytokine expression, this correlation was not evident in controls with elevated cytokine expression [6]. This suggests that the peripheral inflammatory process may reflect altered brain and behaviour in people with schizophrenia, more strongly than in controls. Thus, in controls the tissue targeted by inflammation relating to elevated peripheral cytokines may be outside the brain. Indeed, some individuals will have increased inflammation due to a variety of causes (e.g. *minor* infections, allergies, asthma, arthritis and autoimmunity). Thus, the combination of overt brain dysfunction (such as psychiatric symptoms) and peripheral inflammation may be informative of a subtype of schizophrenia, but will not likely be used as a blood screen to simply diagnose schizophrenia in isolation of the clinical interview or patient history. However, as there are many thousands of inflammation-related molecules that can be measured in the blood, some further assessment as to which molecules are most reliable and informative of this putative subtype, the “inflammatory biotype”, of people with schizophrenia is needed.

There have been a number of studies identifying changes in cytokine proteins in the peripheral blood of prodromal, first episode, acutely ill and chronically ill people with schizophrenia. Brian Miller and colleagues performed a meta-analysis combining data from 40 studies which demonstrated increases in peripheral protein levels of eight pro-inflammatory cytokines [Interleukin (IL)-1, IL-6, IL-8, IL-12, tumour necrosis factor alpha (TNFα), transforming growth factor beta (TGF-β), interferon gamma (IFNγ), soluble IL-2 receptor (sIL2R)] and decreases in two anti-inflammatory cytokines (IL-2 and IL-10) as well as an increase in IL-1 receptor antagonist (IL-1RA) in chronically ill people with schizophrenia and in people with first episode psychosis compared to healthy controls [12]. While they showed that the levels of three cytokines, IL-1β, IL-6 and TGF-β, appear to decrease with antipsychotic treatment, it is likely that these cytokines remain elevated compared to controls as increases in cytokines are typically found in chronically medicated patients [13] as well. A recent paper from the North American Prodrome Longitudinal Study (NAPLS), assessing people at high-risk for schizophrenia showed that the combination of increases in peripheral blood levels of several interleukins (IL-1, IL-7 and IL-8) and molecules capable of modulating the blood-brain barrier function can be used to predict conversion to psychotic illness [14]. Additionally, blood levels of these molecules correlated with the degree of positive symptoms (delusional ideas), cognitive deficits in attention and an increase in dysphoric moods [14]. While increased inflammatory signals were also found in the context of changes in markers of stress axis dysregulation, these results suggest that defining individuals with elevated peripheral cytokines may be useful to identify those that are at high risk of proceeding to worsening psychopathology.

We recently reported that cytokine mRNA transcripts derived from peripheral blood may be used to identify a subset of people with chronic schizophrenia with elevated inflammation [6]. However, how these mRNA measures correspond to the more commonly measured changes in peripheral cytokine proteins has not been demonstrated in people with schizophrenia. Indeed, some studies suggest that measuring (cytokine) mRNA expression from white blood cells may not correlate with changes in circulating protein measurements from plasma and serum [15–17]. Further, since there are distinct molecular levels at which cytokine levels can be measured, one at the steady-state mRNA level and one at the steady-state protein level, each requiring different collection and assay methods, comparative data on each measure would help to prioritise the collection methods of ongoing and future studies in psychiatric populations. Cytokines selected in this study were based on published findings showing changes in specific cytokines in schizophrenia by our group and others [2, 6, 12].

In the present study, we measured inflammation-associated peripheral cytokine mRNA levels together with protein levels in the same cohort of people with schizophrenia compared to healthy controls. We aimed to (1) test for schizophrenia associated peripheral cytokine changes, (2) identify a subset of people with schizophrenia who display increased inflammation based on cytokine mRNA transcripts derived from white blood cells and (3) examine if these inflammatory subgroups show similar patterns of change in peripheral protein levels in both serum and plasma for a range of pro- and anti-inflammatory cytokines. We hypothesised that pro-inflammatory cytokines may be elevated and the anti-inflammatory IL-2 may be reduced in schizophrenia compared to healthy controls. We expected to identify a subgroup of people with elevated cytokine expression by cluster analysis, and we hypothesised that this subgroup would have proportionally more people with schizophrenia than healthy controls. We hypothesised that cytokine protein changes in serum and/or plasma may be reflective of an elevated inflammatory subset as defined by elevated cytokine mRNA expression.

## Methods

### Participants

Ninety-seven people diagnosed with schizophrenia or schizoaffective disorder were recruited from sites in Sydney and Adelaide, Australia, via either clinician or self/family referral following a nationally televised documentary. All patients were living in the community and had been receiving antipsychotic medication for at least 1 year prior to entry in the study. Patient symptom severity scores revealed that the patients displayed mild-to-moderate symptom severity. While the primary purpose of recruitment was for enrolment in a double-blind, placebo-controlled, cross-over trial of adjunctive raloxifene [18], measures taken at baseline prior to commencement of the trial were used for analysis in the current study. To create a control group of unaffected individuals for our case-control study, 87 healthy controls were concurrently recruited through advertisements at the University of New South Wales and the local community in Sydney and Adelaide Australia. We obtained blood samples for RNA extraction from 90 patients and 75 controls (Table 1). Plasma and serum was measured from a subset of participants used for the mRNA study (*n* = 85 and 78 patients, respectively, and *n* = 71 and 65 controls, respectively).

**Table 1:** Cohort demographics

	Full cohort			Cytokine groups				
Demographic	Control (*n* = 75)	Schizophrenia (*n* = 90)	Difference	Con low (*n* = 46)	Con elevated (*n* = 22)	SCZ low (*n* = 43)	SCZ elevated (*n* = 39)	Difference
Age in years (median + range)	29 (20–50)	35 (20–51)	U = 2339, *p* < 0.001	28 (20–50)	31 (22–49)	35 (20–51)	35 (20–50)	*H(3) = 13.41, p < 0.01*
Gender	36 F:39 M	37 F:53 M	χ^2^ = 0.787, *p* = 0.375	20 F:26 M	13 F:9 M	19 F:24 M	16F:23 M	χ^2^ = 2.059, *p* = 0.560
RIN ± SD	7.89 ± 1.11	7.61 ± 1.00	U = 2666.5, *p* = 0.028	8.23 ± 0.60	7.79 ± 1.26	7.81 ± 0.67	7.42 ± 1.18	*H(3) = 8.55, p = 0.036*
Age of onset (in years and range)		22.7 (12–46)				22.5 (12–46)	22.8 (16–32)	U = 920, *p* = 0.448
Duration of illness in years		13.0 ± 7.51				12.7 (3–29)	13.8 (2–31)	U = 932.5, *p* = 0.382
PANSS positive		15.2 ± 4.56				15.0 ± 4.49	15.2 ± 4.43	t(80) = −0.187, *p* = 0.852
PANSS negative		14.4 ± 6.13				14.8 ± 5.70	13.7 ± 6.55	U = 714, *p* = 0.247
PANSS general		30.7 ± 8.80				30.3 ± 8.1	30.7 ± 9.3	U = 829, *p* = 0.930
PANSS total		60.3 ± 16.70				60.2 ± 15.4	59.6 ± 17.7	U = 796, *p* = 0.693
Mean daily chlorpromazine equivalent dose (mg)		558 ± 474				546 ± 489	569 ± 501	U = 834, *p* = 0.967
Body mass index		30.9 ± 6.46				30.4 ± 6.55	30.9 ± 6.41	t(69) = −0.341, *p* = 0.734

Notes: All values, except age, are *mean ± SD* standard deviation, *PANSS* Positive and Negative Symptom Scale, *RIN* RNA integrity number

Antipsychotic doses were obtained from the treating physician and converted to mean daily chlorpromazine equivalent dose [19, 20]. For 78 patients, height and weight was collected for body mass index (BMI) calculation. Diagnostic status was determined using the Structured Clinical Interview for DSM-IV-TR (Diagnostic and Statistical Manual of Mental Disorders 4, text revision) [21] administered by a trained clinician and independently confirmed by another physician.

The Positive and Negative Syndrome Scale (PANSS) was administered by trained psychologists or psychometricians to all patients to obtain measures of positive, negative, general psychopathology and total symptom severity [22]. Patients with a concurrent DSM-IV Axis I psychiatric diagnosis, a history of substance abuse or dependence (within the past 5 years), head injuries with loss of consciousness, seizures, central nervous system infections, uncontrolled diabetes or hypertension, or mental retardation were excluded. Women were excluded if they were currently pregnant. Exclusion criteria for the control group included a personal history of, or a first-degree relative with a DSM-IV Axis I psychiatric diagnosis, history of substance abuse or dependence (within the past 5 years), head injuries with loss of consciousness, seizures, central nervous system infections, uncontrolled diabetes or hypertension, pregnancy, or mental retardation.

Prior to participation in the study, procedures were explained and written informed consent was obtained from all participants. The study was approved by the South Eastern Sydney and Illawarra Area Health Services (07-259), the University of New South Wales Human Research Ethics Committees (07-121 and 09-187) and the Queen Elizabeth Hospital Ethics and Human Research Committee, Adelaide (2010188).

### Sample collection and preparation

Peripheral blood was collected from all participants, in the morning between 9 and 11 am in 9 mL acid citrate dextrose (ACD-B) tubes (BD Biosciences, North Ryde, New South Wales, Australia), 8 mL serum-separating tubes (SST) (Vacutainer, Becton Dickinson, Franklin Lakes, NJ, USA) and 9 mL ethylenediaminetetraacetic acid (EDTA) tubes (Vacuette Vacutainer, Greiner Bio-One, Kremsmünster, Austria).

Total RNA was extracted from white blood cells using the Trizol method (Invitrogen, Carlsbad, CA, USA), and cDNA was synthesised with the Invitrogen Superscript III kit (Invitrogen, Carlsbad, CA, USA) as previously described [23]. Plasma was collected from EDTA tubes via centrifugation at 4 °Celsius for 15 min at 2000 x *g*. SST tubes were incubated at room temperature for 30 min to allow the blood to clot. Upon clotting, the serum was then collected via centrifugation at 4 °Celsius for 5 min at 2000 x *g*. Plasma and serum were aliquoted into protein low-binding tubes (Eppendorf, Hamburg, Germany) and were stored at − 80 °C.

### Quantitative real-time PCR

Transcript levels were measured by quantitative PCR using Applied Biosystems’ 7900HT Real-time PCR system (Foster City, CA, USA). Pre-designed Taqman Gene Expression Assays; Interleukin-1β (Hs01555410_m1), Interleukin-2 (Hs00174114_m1), Interleukin-6 (Hs00174131_m1), Interleukin-8 (HS00174103_m1) and Interleukin-18 (Hs01038788_m1) (Applied Biosystems, Foster City, CA, USA) were used to quantify expression of the cytokine genes. Three housekeeper genes; Peptidylprolyl isomerase A (Hs99999904_m1), TATA box binding protein (Hs00427620_m1), and ubiquitin C (Hs00824723_m1) and its corresponding geometric mean was used for normalisation of the data.

### Data analyses

All analyses were done using the Statistical Package for Social Sciences (version 23, IBM, Armonk, NY, USA) or GraphPad Prism (version 6.04 La Jolla, CA, USA). The alpha was set to 0.05 for all analyses.

### Protein assays

Cytokines were assayed using a Luminex Magpix-based assay (Luminex corporation, Austin, TX, USA). Eight cytokines (IFNγ, TNFα, IL-1β, IL-2, IL-6, IL-8, IL-10 and IL-12 from the Human High Sensitivity T-Cell panel (HST-CYTOMAG60SK, Merck Millipore, Billerica, MA, USA) were analysed, IL-18 was not compatible with the other cytokine assays in this panel and was not measured at the protein level. Serum and plasma samples were thawed at 4 °C and were centrifuged at 1400*g* to remove any aggregate protein that may potentially obstruct the measurement. The supernatant was then transferred to a fresh tube and was diluted 1:2 in assay buffer. A 10-point standard curve with serial dilutions of 1:4 was generated using reconstituted stock standards supplied by the manufacturer; quality controls supplied by the manufacturer were also used to determine assay accuracy. The data was generated using the Millipore Analyst Software (Merck Millipore, Billerica, MA, USA), which calculated average values against a 5-parameter logistic standard curve corrected by background readings. Plasma samples were assayed in duplicate. The average coefficient of variance for duplicate values across analytes for plasma was 0.75%. In plasma, the coefficient of variance in the internal controls across all plates was 52.55%. Having found the coefficient variance between duplicate measures in plasma to be minimal (< 2%) relative to the inter-plate variance, we decided to maximise the number of samples per plate by measuring serum samples without duplicate values. In serum, the coefficient of variance in the internal controls across all plates was 24.03%. The average minimum detectable value across all plates was 0.14 pg/mL for IFNγ, 0.28 pg/mL for IL-10, 0.10 pg/mL for IL-12, 0.23 pg/mL for IL-1β, 0.10 pg/mL for IL-2, 0.05 pg/mL for IL-6, 0.40 pg/mL for IL-8 and 0.12 pg/mL for TNFα.

#### mRNA measures

Separate gene expression experiments were performed for the cohort of 43 controls and 43 patients [6] and for the additional 32 controls and 47 patients who entered the same study at a later time point. In order to analyse these separately run gene expression experiments as a combined cohort, a delta delta Ct (2^−ΔΔCt^) analysis was performed on cycle threshold (Ct) values of all measured cytokines for both experiments [24, 25]. The Ct geometric mean of the three housekeeper genes was used as the internal reference. The average ΔCt of the controls in the first experiment (*n* = 41) and the average ΔCt of the controls in the second experiment (*n* = 36) were used as the calibrators. PCR efficiency across all genes averaged 87.1% ± 6.1%.

Protein concentrations and ΔΔCt mRNA expression values were tested for normality (Shapiro-Wilk) and homogeneity of variances (Levene’s test), IL-6 and IL-8 protein levels in the serum were log transformed to achieve normality. If normality could not be achieved through (log) transformation, non-parametric test were used for analyses. Variables that may contribute to mRNA levels (age, sex and RIN) were included as covariates to the GLM (ANCOVA), but were not found to be significant (*p* > 0.05) and were removed again.

To identify inflammatory subgroups based on cytokine mRNA expression, a recursive two-step cluster analysis was performed on the entire cohort. Since we were interested in defining subgroups with increased inflammation and IL-2 is (mainly) an anti-inflammatory cytokine, we excluded IL-2 from the clustering analysis. Any missing values were replaced by an expectation maximisation (EM) algorithm for the four pro-inflammatory cytokines. Only individuals with gene expression data on at least three out of four pro-inflammatory markers were included in the EM algorithm, others were removed (1 patient, 3 controls). Cytokine values > 2 standard deviations from the group mean were considered outliers. Individuals were excluded from clustering if two or more cytokines were outliers (7 patients, 4 controls). The clustering was performed with 68 controls and 82 patients (Table 1). The resulting model of three clusters had an overall model quality (Silhouette measure) of 0.5, all four variables (IL-18, IL-1β, IL-6, IL-8 in order of contribution) significantly contributed to the model (≥ 0.33 on a scale from 0.1–1.0). To test our hypothesis that people with schizophrenia were more likely to be in the elevated cytokine group than healthy controls, a one-sided Fisher exact test was performed between the low and elevated cytokine groups for people with schizophrenia and healthy controls. Bootstrapping of the cluster algorithm was performed to test the robustness of our clusters. Clustering was performed 10,000 times on subsamples of 135 (*n* - 15) subjects, randomly selected without replacement.

#### Protein measures

A minimum detectable value replacement was performed for data points below the respective minimal detectable value on all protein analytes (on average, 7 data points per analyte), with the exclusion of plasma TNFα and IL-1β (which had a higher degree of missing data). TNFα performed poorly in the plasma and had to be excluded. Only 41% (*n* = 64) of IL-1β samples in the plasma could be detected, plasma IL-1β was included in further analyses but without minimum detectable value replacement (undetectable values were spread proportionally across the different groups).

Percentage difference of all groups compared to low cytokine controls was calculated for all cytokine-mRNA expression levels, plasma protein concentrations and serum protein concentrations, values > two standard deviations from the group ((1) low cytokine control, (2) elevated cytokine control, (3) low cytokine schizophrenia and (4) elevated cytokine schizophrenia) means were excluded. As no significant covariates (age, sex) were identified, differences between groups were tested with a Kruskal-Wallis test for non-parametric data with a correction for multiple comparisons (Bonferroni). For the schizophrenia subgroups, we tested for differences in age of onset, duration of illness, BMI, symptom severity (PANSS) and antipsychotic dose (chlorpromazine equivalent) between the low and elevated cytokine groups.

## Results

### Diagnostic differences in blood cytokine measures

**Fig. 1:**
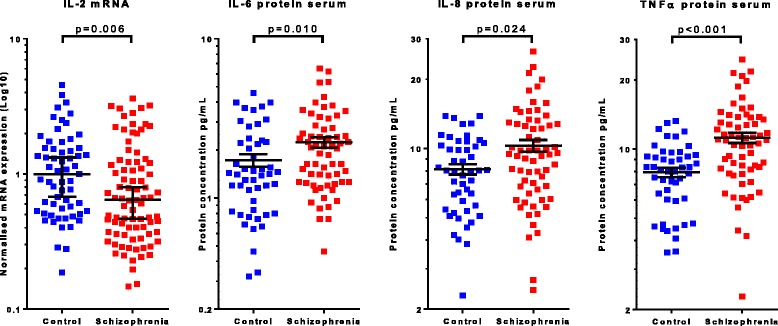
Diagnostic differences in peripheral cytokine expression and protein concentrations. IL-2 mRNA expression level (ΔΔCt) in leukocyte cells is significantly decreased in schizophrenia (*p* = 0.006). There are significant increases in serum protein concentration for the cytokines IL-6 (*p* = 0.010), IL-8 (*p* = 0.024) and TNFα (*p* < 0.001) in schizophrenia. Bars represent the median with 95% confidence interval (IL-2) or the mean with standard error of mean (IL-6, IL-8 and TNFα)

We found that IL-2 mRNA (ΔΔCt) was significantly decreased in people with schizophrenia (median = 0.64) as compared to healthy controls (median = 1.00), *U* = 1898, *p* = 0.006 (Fig. 1). No further significant diagnostic differences in cytokine mRNA levels were found (all *p* > 0.05).

Overall, serum IL-6 protein levels were significantly increased (t(110) = − 2.62, *p* = 0.010) in people with schizophrenia (mean = 2.24 pg/ml, SD = 1.32) compared to healthy controls (mean = 1.73 pg/ml, SD = 1.08) (Fig. 1). Similar results were found for IL-8 (t(110) = −2.92, *p* = 0.024) and TNFα (*U* = 799.5, *p* < 0.001) proteins in the serum which were both significantly higher in people with schizophrenia (IL-8 mean = 10.29 pg/ml, SD = 4.88; TNFα median = 10.94 pg/ml) than in healthy controls (IL-8 mean = 8.13 pg/ml, SD = 2.93; TNFα median = 8.08 pg/ml) (Fig. 1). No further significant differences were found in the protein levels of IFNγ, IL1β, IL-2, IL-10 or IL-12 in either serum or plasma when comparing diagnostic groups (all *p* > 0.05).

### Defining the high inflammatory biotypes using mRNA clustering

**Fig. 2:**
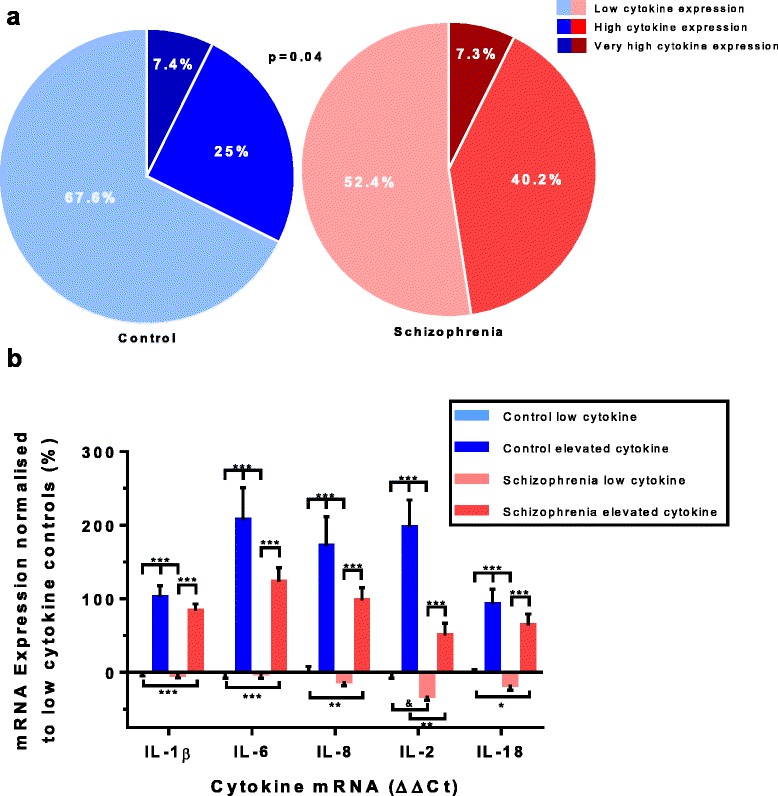
Inflammatory clustering based on peripheral cytokine expression levels. A recursive two step cluster analysis with mRNA expression levels (ΔΔCt) of the cytokines IL-18, IL-1β, IL-6 and IL-8 (in order of contribution) yielded 3 subgroups. (**a**). Increased differences in levels of cytokine expression as compared to low cytokine controls were found for the elevated cytokine group (encompassing groups with high and very high cytokine expression) for all cytokines, regardless of diagnosis (**b**). Bars represent the mean ± standard error (^*&*^ *p* < 0.10, *p < 0.05, **p < 0.01, ***p < 0.001)

The recursive two-step cluster analysis used to define subgroups of people based on pro-inflammatory cytokine mRNA levels yielded three inflammatory subgroups. Cluster 1 (*n* = 89) had a below median expression across all four pro-inflammatory cytokines (IL-1β, IL-6, IL-8, IL-18) and was termed “low cytokine expression”. Cluster two (*n* = 50) had above median expression for two cytokines (IL-18, IL-8) and above third quartile expression for the other two cytokines (IL-1β, IL-6), this cluster was termed “high cytokine expression”. The third cluster (*n* = 11) had above third quartile expressions for all four pro-inflammatory cytokines, all higher than the second cluster and this subgroup was termed “very high cytokine expression”. For further analysis, we combined cluster two and three into one group termed “elevated cytokine expression” and compared this group to the low cytokine expression group. Almost half (47.6%) of the people with schizophrenia were in the elevated cytokine expression group compared to nearly one third (32.4%) of healthy controls with elevated cytokine expression. People with schizophrenia were significantly more likely (*p* = 0.042, Fisher’s exact test) to be in the elevated cytokine expression group than healthy controls (Fig. 2a).

No differences between low and elevated cytokine expression subgroups were found within schizophrenia for age of disease-onset, duration of illness, symptom severity (PANSS), antipsychotics dose (chlorpromazine equivalent), BMI or gender (Table 1).

The elevated cytokine groups showed significant (all *p* ≤ 0.001, Kruskal-Wallis test with Bonferroni correction) increases compared to the low cytokine groups for all tested cytokine mRNAs regardless of diagnosis [IL-1β (H(3) = 73.308, *p* < 0.001), IL-2 (H(3) = 47.499, *p* < 0.001), IL-6 (H(3) = 51.922, *p* < 0.001), IL-8 (H(3) = 46.788, *p* < 0.001), IL-18 (H(3) = 34.758, *p* < 0.001)] (Fig. 2b). When compared to the low cytokine control group, cytokine expression levels were increased by 154% in the elevated cytokine control group, decreased by 13% in the low cytokine schizophrenia group and increased by 85% in the elevated cytokine schizophrenia group. IL-2 also showed a difference between elevated cytokine controls and elevated cytokine schizophrenia subgroups, with schizophrenia patients being significantly (*p* = 0.007) lower than controls by 49%. A trend towards a significant difference was found between low cytokine controls and low cytokine schizophrenia in IL-2 mRNA expression (*p* = 0.072) (Fig. 2b). No further effect of inflammatory subgroup was found for any of the other cytokines. See Additional file 1: Table S1 for a summary of all cytokine mRNA results.

**Fig. 3:**
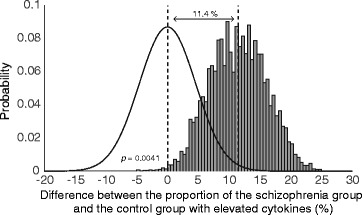
Bootstrapping results of cytokine clustering. Bootstrapped probability distribution of the difference between the schizophrenia and control group with elevated cytokines (grey bars). For comparison, a probability distribution function with equal standard deviation, representing no difference between the groups is presented next to the actual distribution (solid black line). The cumulative probability of people with schizophrenia being similarly or less represented in the elevated inflammation clusters is 0.004. The mean increase in the proportion of the schizophrenia group with elevated cytokines compared with the control group was 11.4% ± 4.6

Using bootstrapping to test the robustness of our cluster analysis, people with schizophrenia were more likely than healthy controls to be in the elevated cytokine expression group (*p* = 0.004) (Fig. 3). However, the mean increase in the proportion of people with schizophrenia with elevated cytokines compared to healthy controls seemed modest at 11.4% ± 4.6.

### Cytokine protein levels in the cytokine transcript defined inflammatory subgroups

**Fig. 4:**
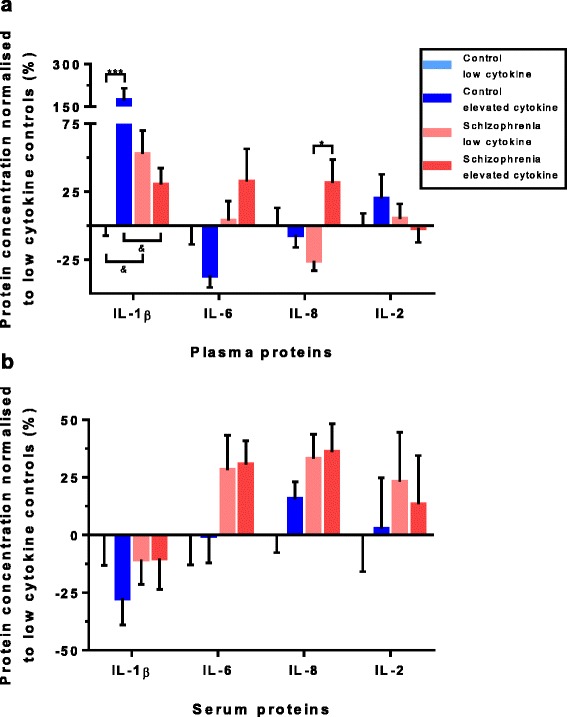
Cytokine protein levels per cytokine cluster. Protein concentrations normalised to the low cytokine controls for the cytokines IL-1β, IL-6, IL-8 and IL-2 (IL-18 was not measured in the protein), showing distinct pattern differences of protein difference levels between plasma (**a**) and serum (**b)**. Significant differences were found for plasma IL-1β, between low and elevated controls (*p* = 0.001) and for plasma IL-8, between low and elevated schizophrenia patients (*p* = 0.047). Bars represent the mean ± standard error (^*&*^ *p* < 0.10, *p < 0.05, ***p < 0.001)

There was an overall effect of mRNA cytokine expression group on IL-1β protein levels in the plasma (H(3) = 16.48, *p* = 0.001). We found a significant increase in IL-1β in elevated cytokine controls compared to low cytokine controls [173% (*p* = 0.001), pairwise comparisons with adjusted *p* values for multiple comparisons], no other subgroup comparisons within the IL-1β protein analyses were significantly different (Fig. 4a). A borderline overall effect of cytokine expression group was also found for IL-8 protein. IL-8 levels in the plasma (H(3) = 7.76, *p* = 0.051) showed a significant 78% increase in the elevated cytokine schizophrenia subgroup as compared to the low cytokine schizophrenia subgroup (*p* = 0.047) (Fig. 4a, pairwise comparisons with adjusted *p* values).

**Fig. 5:**
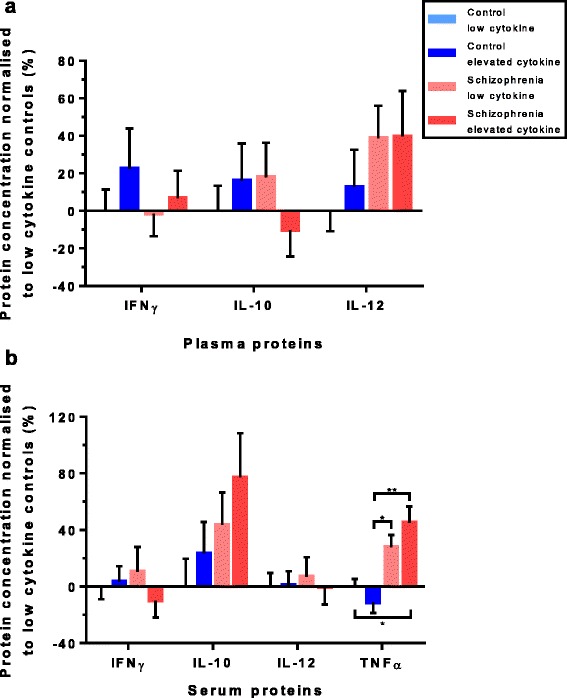
Other cytokine protein levels per cytokine cluster. Protein concentrations normalised to the low cytokine controls for the cytokines IFNγ, IL-10 and IL-12 showed no effect of cytokine cluster groups in the plasma (**a**) or the serum (**b**). For TNFα (serum only), significant differences were found between low cytokine control and elevated cytokine schizophrenia groups (*p* = 0.016), the elevated cytokine control and low cytokine schizophrenia groups (*p* = 0.015) and between the elevated cytokine control and the elevated cytokine schizophrenia group (*p* = 0.002) (**b**). Bars represent the mean ± standard error (*p < 0.05, **p < 0.01)

In contrast to the plasma, no effects of elevated cytokine expression subgroup on cytokine (IL-1β and IL-8) protein levels were found in the serum (Fig. 4b). No cytokine subgroup difference was detected for IFNγ, IL-10 and IL-12 protein levels in the plasma (Fig. 5a) or the serum (Fig. 5b). However, there was a significant main effect of cytokine expression subgroup on TNFα protein levels in the serum (H(3) = 18.36, *p* < 0.001). Pairwise comparisons with adjusted *p* values showed significant increases between low cytokine controls and elevated cytokine schizophrenia of 45% (*p* = 0.016), increases between elevated cytokine controls and low cytokine schizophrenia of 44% (*p* = 0.015) and increases between elevated cytokine controls and elevated cytokine schizophrenia of 64% (*p* = 0.002). This shows TNFα was increased in both the elevated and low cytokine subgroups of people with schizophrenia in serum (Fig. 5b). See Additional file 1: Table S1 for a summary of all protein percentage difference results.

### Across level cytokine measures

**Fig. 6:**
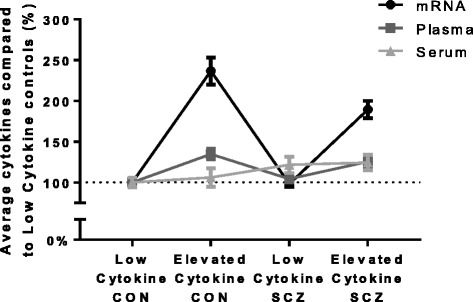
Average cytokines in mRNA, plasma and serum per cytokine cluster. Average pro-inflammatory cytokines expression (mRNA) and concentration (plasma, serum) levels compared to low cytokine controls for the four cytokines (IL-1β, IL-6, IL-8 and IL-2) measured by mRNA expression and protein concentrations. In elevated cytokine controls; mRNA was 144% higher, the plasma proteins were 35% higher and proteins were 6% higher in serum. In low cytokine schizophrenia; mRNA was 11% lower, plasma protein were 4% higher and serum proteins were 22% higher. In elevated cytokine schizophrenia, mRNA was 89% higher, plasma proteins were 25% higher and serum proteins were 25% higher

We measured four cytokines (IL-1β, IL-2, IL-6 and IL-8) using three different methods (mRNA expression, protein levels in serum and protein levels in plasma). All three levels showed distinct patterns of change as visualised in Figs. 2b and 4. The average cytokine expression of the four cytokines measured across mRNA and protein in serum and plasma, relative to the levels of the low inflammation control group, is graphed in Fig. 6. As compared to low cytokine controls; in elevated cytokine controls on average the mRNA expression for the four cytokines (IL-1β, IL-2, IL-6 and IL-8) was 144% higher, the plasma cytokine proteins were 35% higher and cytokine proteins were 6% higher in the serum. In the low cytokine schizophrenia subgroup, mRNA expression was 11% lower on average; cytokine proteins were 4% higher in the plasma and 22% higher in the serum for the four cytokines compared to the low control subgroup. In the elevated cytokine schizophrenia subgroup, cytokine mRNAs were 89% higher; cytokine proteins were 25% higher in plasma and 25% higher in serum for the four cytokines as compared to low cytokine controls. As may be expected from inspection of Fig. 6, no significant correlations were found between mRNA expression and protein levels in either the serum or the plasma within individual cytokines. However, significant Spearman’s rho correlations (correlation coefficients = 0.217–0.644, *p* = 0.018– < 0.001) were found between protein levels in the serum and plasma for IL-1β, IL-2, IL-6 and IL-8. Similarly, we found significant Spearman’s rho correlations (correlation coefficients = 0.534–0.600, all *p* < 0.001) between protein levels in the serum and plasma for the three cytokines (IL-10, IL-12 and IFNγ) that were not measured by mRNA (Additional file 2: Table S2).

## Discussion

In this study, we found a lower level of mRNA for the anti-inflammatory cytokine IL-2 in the blood of people with schizophrenia. We have also shown higher levels of three pro-inflammatory cytokines IL-6, IL-8 and TNF-α in the serum of people with schizophrenia, indicating a role of moderate chronic inflammation in schizophrenia. An increase in occurrence of the “elevated cytokine subgroup” in schizophrenia was found by measuring transcript levels of cytokines from white blood cells. When comparing our clustering of individuals based on mRNA from blood to clustering of individuals based on mRNA from brain [2, 3] overall more people fall into the elevated cytokine biotype based on the blood mRNA expression. We did find quite a high proportion of controls who were also classified as having “elevated cytokines” and consequently, the difference between the proportion of people with schizophrenia and healthy controls that fall into the elevated cytokine subgroup is smaller when assayed in blood than in brain. Nonetheless, our study provides further evidence that peripheral cytokine mRNA levels could be used to identify a subgroup of people with schizophrenia who may be in the elevated inflammation biotype [6, 2].

Overall, in people with schizophrenia, we found a lower mRNA expression level of IL-2, believed to be primarily an anti-inflammatory cytokine, supporting earlier findings of decreased IL-2 protein in serum/plasma of people with schizophrenia [11]. Thus, it may be that people with schizophrenia have a blunted ability to dampen or attenuate unwanted inflammation via producing less IL-2. IL-2 was once thought to activate T cells, but has recently been identified as critical in activation-induced cell death (AICD) of autoreactive T cells [26]. Our finding of lower levels of IL-2 mRNA expression in people with schizophrenia may also suggest that in schizophrenia more T cells escape this important regulatory step and thus, people with schizophrenia may be expected to have more self-reactive T cells. Lower levels or lower function of IL-2 may be at least a partial explanation of why more brain autoantibodies can be found in blood and cerebrospinal fluid of people with schizophrenia [27].

In this study, we did not reproduce our finding of upregulated peripheral IL-1β mRNA expression in the overall group of people with schizophrenia, previously reported in a subset of this cohort [6]. However, in this analysis we may have introduced some variance by assaying subjects at two different time points, so failure to find overall increases should be taken with caution. IL-1β is considered a master regulator of neuroinflammation [28], and in this cohort, high levels of IL-1β mRNA correlated with increased protein levels of IL6 and TNF alpha. Additionally, IL-1β expression was informative in the cluster analysis to identify the high inflammatory biotype and tended to correspond with high levels in other cytokine mRNA (data not shown) as found before [6] confirming IL-1β as a major player in inflammation.

In our analysis of protein levels of cytokines, we found significant increases in serum protein levels for the cytokines IL-6, IL-8 and TNFα in the whole group of people with schizophrenia compared to the whole group of healthy controls. TNFα has recently been reported to also be upregulated in the plasma of people with schizophrenia [29]. Since peripheral IL-6 and TNFα are considered to be mainly macrophage-derived cytokines [30], this suggests an over-activity of monocytes capable of transmigrating into tissue differentiating as macrophages and phagocytosing cellular and subcellular substrates in tissues, possibly the brain, in people with schizophrenia. In brain, we and others find an upregulation of IL-6 and IL-8 mRNA expression in the dorsal lateral prefrontal cortex (DLPFC) of people with schizophrenia compared to controls [2, 4], but no change in TNFα [3, 29]. In the brain, the resident immune cells or microglia can also produce these pro-inflammatory cytokines and several recent studies have identified increased activated microglial binding with the PK11195 ligand in the brains of people with schizophrenia compared to healthy controls [31–33] corresponding to the increase in microglia density found by direct immunohistochemical measures in post-mortem brains by our group [2] and others [34, 35]. A recent study by Bloomfield et al. used the PBR28 ligand to monitor microglia activity in individuals in two cohorts, (1) a neuroleptic naïve group at ultra-high risk of developing schizophrenia and (2) a group with chronic schizophrenia [36]. Importantly, they found increased binding in both cohorts suggesting that changes in microglia may be found throughout the course of schizophrenia. Furthermore, increases in microglial binding in those at high risk of developing psychosis were highly correlated with positive symptoms [36] similar to the findings reported in the NAPLS study using blood biomarkers of inflammation [14]. These studies suggest that increases in brain microglia are most likely not secondary to antipsychotic medications, and this proposition is further supported by clinical work showing that antipsychotics decrease cytokine expression [37–39]. However, a few other studies suggest that microglial activation via positon emission tomography (PET) is not easily found at various stages of schizophrenia [40–42].

Thus, the work to date suggests that neuroinflammation is a part of the pathophysiology of schizophrenia, may be directly related to the experience of psychosis and may be able to be targeted with treatment before the first full blown episode of psychosis emerges. However, since other imaging studies are suggesting that there may be no change or even a reduction in the microglia-related signal in some people with schizophrenia [1], further studies into microglia in the schizophrenic brain are required. Our work suggests that increases in blood cytokines, especially in IL-6, IL-8 and TNFα, persist beyond early psychosis and can be found in chronic patients even when stabilised on antipsychotic medications.

Previously, we have reported that ~ 40% of people with schizophrenia are in the high inflammatory biotype in the blood and brain compared to 20% of healthy controls in peripheral blood [6] and 10% of healthy controls in post-mortem prefrontal cortex tissue [2]. Here, we observe a proportion of 48% of people with schizophrenia having elevated peripheral cytokine expression levels as compared to 32% of healthy controls, showing slightly higher proportions of people with the elevated cytokine biotype in both people with schizophrenia and healthy controls. Bootstrapping demonstrated that this difference in the proportion of people with schizophrenia with the elevated cytokine biotype relative to healthy controls is statistically significant, but likely smaller than the 16% reported in our primary analysis with an estimated mean difference of ~ 11%. This difference in this clinical cohort based on measurements from blood is smaller than the percentage difference in the elevated cytokine subgroups from the perspective of measuring cytokine mRNA in the post-mortem brain. This smaller difference may be partly explained by the many sources for blood cytokines related to inflammation in a range of peripheral tissues. Our results support the general concession that elevated blood cytokines are not specific to schizophrenia. Although not significant for all cytokines in this study, the mRNA expression seems to in fact be higher in the elevated control group than in the elevated schizophrenia group; this could indicate that healthy controls are able to mount more of a cytokine response than schizophrenia patients where there may be more of a blunted response to inflammatory triggering events. Within the schizophrenia group, we did not find a clinical difference for any of the measurements for symptom severity (PANSS—positive, negative, general and total) based on cytokine expression subgroup, confirming some other studies indicating that elevated peripheral inflammation does not necessarily correlate with symptom severity in chronically ill patients receiving antipsychotic treatment [43, 44].

While the underlying cause of the cytokine dysregulation we have found here is not known, decreased IL-2 [45], increased IL-6 [46], IL-8 and TNFα [47] have all been associated with *Toxoplasma gondii* infections, of which the seroprevelance is increased in schizophrenia [48]. However, increases in IL-6, IL-8 and TNFα are also associated with a wide variety of immune activity like acute-phase response [49] (e.g. sepsis and viral infections) and autoimmunity [50] which also can be related to schizophrenia [51]. Many other cytokines are associated with the response to viral and bacterial infections for which we did not observe significant changes or were not assessed in this study, making causality of the cytokine dysregulation observed in schizophrenia at this stage speculative. One further possibility is that certain genetic factors may predispose people with schizophrenia towards immune dysregulation as a recent genome-wide association study in schizophrenia identified genes on the human leukocyte antigen locus as one of the strongest associations with schizophrenia [52].

We did not find mRNA expression to correspond with protein levels suggesting that white blood cells may not be the main source of peripheral cytokine proteins. Indeed, a wide range of cells are known to secrete cytokines, including fibroblasts, adipocytes, endothelial cells, neurons [53], heart- [54] and liver [55] cells. Differences between protein concentrations in the serum and plasma, where we found diagnostic differences for IL-6, IL-8 and TNFα in the serum but not the plasma, are most likely caused by the removal of some sources of cytokines in the plasma. In plasma, platelets as well as fibrinogens are largely removed resulting in different protein concentrations and biocompartments as compared to serum [56, 57]. The plasma was centrifuged without clotting, a step that should remove 95% of all platelets [58]. Platelets can produce and store cytokines and other mediators of inflammation in α-granules [59, 60]. This suggests that people with schizophrenia in our cohort may have increased cytokine levels within the blood platelets compared to healthy controls as in this cohort platelet counts were not increased in people with schizophrenia (data not shown).

There are several limitations of our study. The interrelationships between cytokines are common and complex and an inflammatory response triggers a cascade of molecular changes. The present study provides cytokine levels for only one snapshot in time; longitudinal studies are required to study cytokine dysregulations in schizophrenia across the disease course. A possible confound in our study was that all patients in our study were receiving antipsychotics which are known to affect cytokine levels [12]. Further, many of our patients had high BMIs [61] which in turn can cause elevations in blood cytokines through release of cytokines such as IL-6 and TNFα from fat cells [62]. However, antipsychotic doses (chlorpromazine equivalent) and BMI did not differ between normal and elevated cytokine expression groups for people with schizophrenia in our study. In support of our interpretation, other studies suggest that increased incidence of elevated cytokine expression is not just an effect of treatment or BMI. For example, Beumer et al. showed that increases in circulating levels of IL-1β, IL-6 and TNFα are not associated with metabolic syndrome in schizophrenia [30], and Fernandes et al. showed by meta-analysis that CRP is not related to antipsychotic dose in schizophrenia [63]. However, multiple bio behavioural factors are known to have an effect on circulating inflammatory markers [64] including psychological stress [65] and depression [66], which have not been examined in this study. The inter-plate variance of our protein assays, especially in the plasma was quite high. Cytokines are not highly abundant in the periphery of relatively healthy people [67] and most individuals were towards the lower end of detection for these assays, which may have increased the variability of our data. Despite this, our cytokine assays did have reasonable lower limits of detection with relatively few missing data point for most assays.

## Conclusion

We have highlighted that depending on the level of measurement (mRNA, serum- or plasma proteins), the magnitude and even the type of cytokines that are elevated in a subgroup of people with schizophrenia are not consistent. This raises the question whether similar biotypes can be identified when measuring blood proteins instead of mRNA expression levels. Further studies of this and other independent cohorts are required to identify the best panel and measurement level of inflammatory markers in the periphery of people with schizophrenia to identify the elevated cytokine biotype. In the meantime, we advise that it may be important to collect mRNA, serum and plasma levels for comparisons in future studies in schizophrenia research. We suggest that further interrogation of transcriptional changes in white blood cells may help to identify the reasons why peripheral cytokines are elevated in a subset of people with schizophrenia.

## Additional files

Additional file 1: Table S1. Percentage change results. (DOCX 20 kb)

Additional file 2: Table S2. Significant correlations between serum and plasma cytokines. (DOCX 14 kb)

## Abbreviations

ACD-B: Acid citrate dextrose solution B
BMI: Body mass index
CRP: C-reactive protein
DSM: Diagnostic and Statistical Manual of Mental Disorders
EDTA: Ethylenediaminetetraacetic acid
GLM: General linear model
IFNγ: Interferon gamma
IL-(x): Interleukin
IL-1RA: Interleukin-1 receptor antagonist
PANSS: Positive and negative symptom scale
RIN: RNA integrity number
sIL-2R: Soluble interleukin-2 receptor
SST: Serum separating tubes
TGF-β: Transforming growth factor beta
TNFα: Tumour necrosis factor alpha

## References

[1] Trepanier MO, Hopperton KE, Mizrahi R, Mechawar N, Bazinet RP (2016). Postmortem evidence of cerebral inflammation in schizophrenia: a systematic review. Mol Psychiatry.

[2] Fillman SG, Cloonan N, Catts VS, Miller LC, Wong J, McCrossin T, Cairns M, Weickert CS (2013). Increased inflammatory markers identified in the dorsolateral prefrontal cortex of individuals with schizophrenia. Mol Psychiatry.

[3] Fillman SG, Sinclair D, Fung SJ, Webster MJ, Shannon Weickert C (2014). Markers of inflammation and stress distinguish subsets of individuals with schizophrenia and bipolar disorder. Transl Psychiatry.

[4] Volk DW, Chitrapu A, Edelson JR, Roman KM, Moroco AE, Lewis DA (2015). Molecular mechanisms and timing of cortical immune activation in schizophrenia. Am J Psychiatry.

[5] Weickert CS, Weickert TW, Pillai A, Buckley PF (2013). Biomarkers in schizophrenia: a brief conceptual consideration. Dis Markers.

[6] Fillman SG, Weickert TW, Lenroot RK, Catts SV, Bruggemann JM, Catts VS, Weickert CS (2016). Elevated peripheral cytokines characterize a subgroup of people with schizophrenia displaying poor verbal fluency and reduced Broca/'s area volume. Mol Psychiatry.

[7] Mehta D, Raison CL, Woolwine BJ, Haroon E, Binder EB, Miller AH, Felger JC (2013). Transcriptional signatures related to glucose and lipid metabolism predict treatment response to the tumor necrosis factor antagonist infliximab in patients with treatment-resistant depression. Brain Behav Immun.

[8] Raison CL, Rutherford RE, Woolwine BJ (2013). A randomized controlled trial of the tumor necrosis factor antagonist infliximab for treatment-resistant depression: the role of baseline inflammatory biomarkers. JAMA Psychiatry.

[9] Weinberger JF, Raison CL, Rye DB, Montague AR, Woolwine BJ, Felger JC, Haroon E, Miller AH (2015). Inhibition of tumor necrosis factor improves sleep continuity in patients with treatment resistant depression and high inflammation. Brain Behav Immun.

[10] Catts VS, Wong J, Fillman SG, Fung SJ, Shannon Weickert C (2014). Increased expression of astrocyte markers in schizophrenia: association with neuroinflammation. Aust N Z J Psychiatry.

[11] Zhang Y, Catts VS, Sheedy D, McCrossin T, Kril JJ, Shannon Weickert C (2016). Cortical grey matter volume reduction in people with schizophrenia is associated with neuro-inflammation. Transl Psychiatry.

[12] Miller BJ, Buckley P, Seabolt W, Mellor A, Kirkpatrick B (2011). Meta-analysis of cytokine alterations in schizophrenia: clinical status and antipsychotic effects. Biol Psychiatry.

[13] Theodoropoulou S, Spanakos G, Baxevanis CN, Economou M, Gritzapis AD, Papamichail MP, Stefanis CN (2001). Cytokine serum levels, autologous mixed lymphocyte reaction and surface marker analysis in never medicated and chronically medicated schizophrenic patients. Schizophr Res.

[14] Perkins DO, Jeffries CD, Addington J, Bearden CE, Cadenhead KS, Cannon TD, Cornblatt BA, Mathalon DH, McGlashan TH, Seidman LJ (2015). Towards a psychosis risk blood diagnostic for persons experiencing high-risk symptoms: preliminary results from the NAPLS project. Schizophr Bull.

[15] Lin H, Joehanes R, Pilling LC, Dupuis J, Lunetta KL, Ying S-X, Benjamin EJ, Hernandez D, Singleton A, Melzer D (2014). Whole blood gene expression and interleukin-6 levels. Genomics.

[16] Schindler R, Clark BD, Dinarello CA (1990). Dissociation between interleukin-1 beta mRNA and protein synthesis in human peripheral blood mononuclear cells. J Biol Chem.

[17] O'Rourke RW, Kay T, Lyle EA, Traxler SA, Deveney CW, Jobe BA, Roberts CT, Marks D, Rosenbaum JT (2006). Alterations in peripheral blood lymphocyte cytokine expression in obesity. Clin Exp Immunol.

[18] Weickert TW, Weinberg D, Lenroot R, Catts SV, Wells R, Vercammen A, O'Donnell M, Galletly C, Liu D, Balzan R (2015). Adjunctive raloxifene treatment improves attention and memory in men and women with schizophrenia. Mol Psychiatry.

[19] Bollini P, Pampallona S, Nieddu S, Bianco M, Tibaldi G, Munizza C (2008). Indicators of conformance with guidelines of schizophrenia treatment in mental health services. Psychiatr Serv.

[20] Woods SW. Chlorpromazine equivalent doses for the newer atypical antipsychotics. J Clin Psychiatry. 2003;64(6):663-7. 10.4088/jcp.v64n0607 12823080

[21] First MBS, Robert L, Gibbon M, Williams JBW (2002). Structured clinical interview for DSM-IV-TR Axis I disorders, research version, patient edition. (SCID-I/NP).

[22] Kay SR, Fiszbein A, Opler LA. The positive and negative syndrome scale (PANSS) for schizophrenia. Schizophr Bull. 1987;13(2):261-76. 10.1093/schbul/13.2.261 3616518

[23] Weickert CS, Sheedy D, Rothmond DA, Dedova I, Fung S, Garrick T, Wong J, Harding AJ, Sivagnanansundaram S, Hunt C (2010). Selection of reference gene expression in a schizophrenia brain cohort. Aust N Z J Psychiatry.

[24] Livak KJ, Schmittgen TD (2001). Analysis of relative gene expression data using real-time quantitative PCR and the 2−ΔΔCT method. Methods.

[25] Pfaffl MW (2001). A new mathematical model for relative quantification in real-time RT–PCR. Nucleic Acids Res.

[26] Nelson BH (2004). IL-2, regulatory T cells, and tolerance. J Immunol.

[27] Hammer C, Stepniak B, Schneider A, Papiol S, Tantra M, Begemann M, Siren AL, Pardo LA, Sperling S, Mohd Jofrry S (2014). Neuropsychiatric disease relevance of circulating anti-NMDA receptor autoantibodies depends on blood-brain barrier integrity. Mol Psychiatry.

[28] Basu A, Krady JK, Levison SW (2004). Interleukin-1: a master regulator of neuroinflammation. J Neurosci Res.

[29] Hoseth EZ, Ueland T, Dieset I, Birnbaum R, Shin JH, Kleinman JE, Hyde TM, Morch RH, Hope S, Lekva T, et al. A study of TNF pathway activation in schizophrenia and bipolar disorder in plasma and brain tissue. Schizophr Bull. 2017;43(4):881-90. 10.1093/schbul/sbw183 PMC5515106 28049760

[30] Beumer W, Drexhage RC, De Wit H, Versnel MA, Drexhage HA, Cohen D (2012). Increased level of serum cytokines, chemokines and adipokines in patients with schizophrenia is associated with disease and metabolic syndrome. Psychoneuroendocrinology.

[31] van Berckel BN, Bossong MG, Boellaard R, Kloet R, Schuitemaker A, Caspers E, Luurtsema G, Windhorst AD, Cahn W, Lammertsma AA (2008). Microglia activation in recent-onset schizophrenia: a quantitative (R)-[11C]PK11195 positron emission tomography study. Biol Psychiatry.

[32] Doorduin J, de Vries EF, Willemsen AT, de Groot JC, Dierckx RA, Klein HC (2009). Neuroinflammation in schizophrenia-related psychosis: a PET study. J Nucl Med.

[33] Kurumaji A, Wakai T, Toru M (1997). Decreases in peripheral-type benzodiazepine receptors in postmortem brains of chronic schizophrenics. J Neural Transm.

[34] Bayer TA, Buslei R, Havas L, Falkai P (1999). Evidence for activation of microglia in patients with psychiatric illnesses. Neurosci Lett.

[35] Radewicz K, Garey LJ, Gentleman SM, Reynolds R (2000). Increase in HLA-DR Immunoreactive microglia in frontal and temporal cortex of chronic schizophrenics. J Neuropathol Exp Neurol.

[36] Bloomfield PS, Selvaraj S, Veronese M, Rizzo G, Bertoldo A, Owen DR, Bloomfield MAP, Bonoldi I, Kalk N, Turkheimer F (2016). Microglial activity in people at ultra high risk of psychosis and in schizophrenia: an [11C]PBR28 PET brain imaging study. Am J Psychiatry.

[37] Cazzullo CL, Sacchetti E, Galluzzo A, Panariello A, Adorni A, Pegoraro M, Bosis S, Colombo F, Trabattoni D, Zagliani A (2002). Cytokine profiles in schizophrenic patients treated with risperidone: a 3-month follow-up study. Prog Neuro-Psychopharmacol Biol Psychiatry.

[38] Meyer JM, McEvoy JP, Davis VG, Goff DC, Nasrallah HA, Davis SM, Hsiao JK, Swartz MS, Stroup TS, Lieberman JA (2009). Inflammatory markers in schizophrenia: comparing antipsychotic effects in phase 1 of the clinical antipsychotic trials of intervention effectiveness study. Biol Psychiatry.

[39] Xiang Yang Zhang DFZ, Cao LY, Zhang PY, Wu GY, Shen YC. Changes in serum Interleukin-2, −6, and −8 levels before and during treatment with Risperidone and haloperidol: relationship to outcome in schizophrenia. J Clin Psychiatry. 2004;65(7):940-7. 10.4088/jcp.v65n0710 15291683

[40] Collste K, Plaven-Sigray P, Fatouros-Bergman H, Victorsson P, Schain M, Forsberg A, Amini N, Aeinehband S, Karolinska Schizophrenia Project c, Erhardt S, et al. Lower levels of the glial cell marker TSPO in drug-naive first-episode psychosis patients as measured using PET and [lsqb]11C[rsqb]PBR28. Mol Psychiatry. 2017;22(6):850-6. 10.1038/mp.2016.247 28194003

[41] Coughlin JM, Wang Y, Ambinder EB, Ward RE, Minn I, Vranesic M, Kim PK, Ford CN, Higgs C, Hayes LN (2016). In vivo markers of inflammatory response in recent-onset schizophrenia: a combined study using [lsqb]11C[rsqb]DPA-713 PET and analysis of CSF and plasma. Transl Psychiatry.

[42] Kenk M, Selvanathan T, Rao N, Suridjan I, Rusjan P, Remington G, Meyer JH, Wilson AA, Houle S, Mizrahi R (2015). Imaging neuroinflammation in gray and white matter in schizophrenia: an in-vivo PET study with [ 18 F]-FEPPA. Schizophr Bull.

[43] Dickerson F, Stallings C, Origoni A, Boronow J, Yolken R (2007). C-reactive protein is associated with the severity of cognitive impairment but not of psychiatric symptoms in individuals with schizophrenia. Schizophr Res.

[44] Coelho FM, Reis HJ, Nicolato R, Romano-Silva MA, Teixeira MM, Bauer ME, Teixeira AL (2008). Increased serum levels of inflammatory markers in chronic institutionalized patients with schizophrenia. Neuroimmunomodulation.

[45] Hammouda NA, Hegazy IH, Rashwan IA, Ali SM (1998). Effect of interleukin-2 on experimental acute toxoplasmosis. J Egypt Soc Parasitol.

[46] Fischer H-G, Nitzgen B, Reichmann G, Hadding U (1997). Cytokine responses induced by Toxoplasma gondii in astrocytes and microglial cells. Eur J Immunol.

[47] Lahmar I, Abou-Bacar A, Abdelrahman T, Guinard M, Babba H, Ben Yahia S, Kairallah M, Speeg-Schatz C, Bourcier T, Sauer A (2009). Cytokine profiles in toxoplasmic and viral uveitis. J Infect Dis.

[48] Torrey EF, Bartko JJ, Yolken RH (2012). Toxoplasma gondii and other risk factors for schizophrenia: an update. Schizophr Res.

[49] Gabay C, Kushner I (1999). Acute-phase proteins and other systemic responses to inflammation. N Engl J Med.

[50] Gershov D, Kim S, Brot N, Elkon KB (2000). C-reactive protein binds to apoptotic cells, protects the cells from assembly of the terminal complement components, and sustains an Antiinflammatory innate immune response: implications for systemic autoimmunity. J Exp Med.

[51] Benros ME, Waltoft BL, Nordentoft M (2013). Autoimmune diseases and severe infections as risk factors for mood disorders: a nationwide study. JAMA Psychiatry.

[52] Schizophrenia Working Group of the Psychiatric Genomics C (2014). Biological insights from 108 schizophrenia-associated genetic loci. Nature.

[53] Potvin S, Stip E, Sepehry AA, Gendron A, Bah R, Kouassi E (2008). Inflammatory cytokine alterations in schizophrenia: a systematic quantitative review. Biol Psychiatry.

[54] Aoyagi T, Matsui T: The Cardiomyocyte as a source of cytokines in cardiac injury. J Cell Sci Ther 2011, 2012(0):003. 10.4172/2157-7013.s5-003 PMC3594870 23493668

[55] Dong W, Simeonova PP, Gallucci R, Matheson J, Fannin R, Montuschi P, Flood L, Luster MI (1998). Cytokine expression in hepatocytes: role of oxidant stress. J Interf Cytokine Res.

[56] Leng SX, McElhaney JE, Walston JD, Xie D, Fedarko NS, Kuchel GA (2008). Elisa and multiplex technologies for cytokine measurement in inflammation and aging research. J Gerontol A Biol Sci Med Sci.

[57] Lundblad R. Considerations for the use of blood plasma and serum for proteomic analysis. Int J Genomics Proteomics. 2003;1(2).

[58] Chandler WL (2013). Microparticle counts in platelet-rich and platelet-free plasma, effect of centrifugation and sample-processing protocols. Blood Coagul Fibrinolysis.

[59] Blair P, Flaumenhaft R (2009). Platelet α-granules: basic biology and clinical correlates. Blood Rev.

[60] Smyth SS, McEver RP, Weyrich AS, Morrell CN, Hoffman MR, Arepally GM, French PA, Dauerman HL, Becker RC (2009). For the platelet colloquium P: platelet functions beyond hemostasis. J Thromb Haemost.

[61] Wirshing DA, Wirshing WC, Kysar L, Berisford MA, Goldstein D, Pashdag J, Mintz J, Marder SR (1999). Novel antipsychotics: comparison of weight gain liabilities. J Clin Psychiatry.

[62] Fried SK, Bunkin DA, Greenberg AS (1998). Omental and subcutaneous adipose tissues of obese subjects release Interleukin-6: depot difference and regulation by glucocorticoid. J Clin Endocrinol Metab.

[63] Fernandes BS, Steiner J, Bernstein HG, Dodd S, Pasco JA, Dean OM, Nardin P, Goncalves CA, Berk M (2016). C-reactive protein is increased in schizophrenia but is not altered by antipsychotics: meta-analysis and implications. Mol Psychiatry.

[64] O’Connor M-F, Bower JE, Cho HJ, Creswell JD, Dimitrov S, Hamby ME, Hoyt MA, Martin JL, Robles TF, Sloan EK (2009). To assess, to control, to exclude: effects of biobehavioral factors on circulating inflammatory markers. Brain Behav Immun.

[65] Segerstrom SC, Miller GE (2004). Psychological stress and the human immune system: a meta-analytic study of 30 years of inquiry. Psychol Bull.

[66] Zorrilla EP, Luborsky L, McKay JR, Rosenthal R, Houldin A, Tax A, McCorkle R, Seligman DA, Schmidt K (2001). The relationship of depression and stressors to immunological assays: a meta-analytic review. Brain Behav Immun.

[67] de Jager W, Bourcier K, Rijkers GT, Prakken BJ, Seyfert-Margolis V (2009). Prerequisites for cytokine measurements in clinical trials with multiplex immunoassays. BMC Immunol.

