# Supplementary material for: Using blood cytokine measures to define high inflammatory biotype of schizophrenia and schizoaffective disorder
Source: J Neuroinflammation. 2017 Sep 18;14:188. doi: 10.1186/s12974-017-0962-y (PMC5604300; doi:10.1186/s12974-017-0962-y)
Supplement: Supplementary file 1 — Percentage change results. (DOCX 20 kb) [file 12974_2017_962_MOESM1_ESM.docx]

**Additional file 1: Table S1 Percentage change results**

| **Level** | **Cytokine** | **Low Cytokine** | | | **Elevated Cytokine Control** | | | **Low Cytokine Schizophrenia** | | | **Elevated Cytokine Schizophrenia** | | |
| --- | --- | --- | --- | --- | --- | --- | --- | --- | --- | --- | --- | --- | --- |
|  |  | **Control** | | |  |  |  |  |  |  |  |  |  |
|  |  | **Mean** | **SEM** | **n** | **Mean** | **SEM** | **n** | **Mean** | **SEM** | **n** | **Mean** | **SEM** | **n** |
| mRNA | IL-1β | 0 | 4.7 | 45 | 102.2 | 15.9 | 20 | -2.7 | 4.6 | 41 | 83.8 | 9.2 | 37 |
|  | IL-6 | 0 | 8 | 44 | 207 | 44 | 20 | -0.9 | 6.7 | 40 | 123.2 | 19.2 | 36 |
|  | IL-8 | 0 | 8.4 | 44 | 172.4 | 39 | 20 | -12.1 | 6 | 40 | 98.1 | 17.1 | 36 |
|  | IL-2 | 0 | 8.1 | 38 | 197.3 | 37 | 18 | -31.7 | 5.9 | 39 | 50.5 | 16.1 | 36 |
|  | IL-18 | 0 | 4.3 | 45 | 92.5 | 20.4 | 21 | -16.4 | 7.8 | 41 | 63.9 | 15.6 | 37 |
| Plasma | IL-1β | 0 | 7.5 | 16 | 172.9 | 41.5 | 5 | 52.9 | 17.2 | 17 | 30.3 | 12.1 | 14 |
|  | IL-6 | 0 | 13.9 | 34 | -36.8 | 8.7 | 15 | 3.7 | 14.3 | 35 | 32.5 | 24 | 31 |
|  | IL-8 | 0 | 13 | 38 | -7 | 9.1 | 15 | -26.1 | 7.1 | 37 | 31.4 | 17.2 | 31 |
|  | IL-2 | 0 | 9.1 | 37 | 19.9 | 17.8 | 14 | 5.1 | 10.8 | 35 | -1.8 | 10.5 | 29 |
| Serum | IL-1β | 0 | 13.2 | 29 | -27.4 | 11.6 | 20 | -10.6 | 10.7 | 36 | -10.2 | 13.4 | 25 |
|  | IL-6 | 0 | 12.9 | 29 | -0.5 | 11.7 | 20 | 28.3 | 15 | 36 | 30.6 | 10.3 | 27 |
|  | IL-8 | 0 | 7.6 | 29 | 15.7 | 7.4 | 19 | 33.1 | 10.6 | 37 | 36 | 12.3 | 27 |
|  | IL-2 | 0 | 15.8 | 28 | 2.8 | 22.1 | 20 | 23.2 | 21.4 | 37 | 13.4 | 21.2 | 25 |
| Plasma | IFNγ | 0 | 11.5 | 36 | 22.5 | 21.4 | 15 | -1.5 | 12.1 | 35 | 6.9 | 14.5 | 27 |
|  | IL-10 | 0 | 13.4 | 31 | 16.1 | 19.9 | 14 | 18 | 18.2 | 31 | -10.3 | 13.9 | 28 |
|  | IL-12 | 0 | 10.9 | 33 | 12.9 | 19.7 | 13 | 38.7 | 17.4 | 32 | 39.7 | 24.2 | 25 |
| Serum | IFNγ | 0 | 8.9 | 28 | 3.5 | 10.9 | 20 | 10.7 | 17.3 | 37 | -10 | 12 | 27 |
|  | IL-10 | 0 | 19.7 | 27 | 23.6 | 22 | 20 | 43.7 | 22.9 | 35 | 77.1 | 31.3 | 27 |
|  | IL-12 | 0 | 9.6 | 28 | 1.2 | 9.6 | 20 | 7.2 | 13.6 | 36 | -0.4 | 12.3 | 27 |
|  | TNFα | 0 | 5.4 | 28 | -11.2 | 7.5 | 19 | 28 | 8.4 | 37 | 45.3 | 11.4 | 26 |
