# Supplementary material for: Using blood cytokine measures to define high inflammatory biotype of schizophrenia and schizoaffective disorder
Source: J Neuroinflammation. 2017 Sep 18;14:188. doi: 10.1186/s12974-017-0962-y (PMC5604300; doi:10.1186/s12974-017-0962-y)
Supplement: Supplementary file 2 — Significant correlations between serum and plasma cytokines. (DOCX 14 kb) [file 12974_2017_962_MOESM2_ESM.docx]

**Additional file 2: Table S2 Significant correlations between serum and plasma cytokines**

| **Cytokine levels in serum vs. Cytokine levels in plasma** | | | | | | | |
| --- | --- | --- | --- | --- | --- | --- | --- |
|  | IL-1β | IL-2 | IL-6 | IL-8 | IL-10 | IL-12 | IFNγ |
| Correlation Coefficient | 0.217 | 0.644 | 0.544 | 0.297 | 0.583 | 0.600 | 0.534 |
| Sig. (2-tailed) | **0.018** | **<0.0001** | **<0.0001** | **0.001** | **<0.0001** | **<0.0001** | **<0.0001** |
| n | 118 | 116 | 116 | 117 | 114 | 116 | 118 |
